# Hidden biodiversity revealed by integrated morphology and genetic species delimitation of spring dwelling water mite species (Acari, Parasitengona: Hydrachnidia)

**DOI:** 10.1186/s13071-019-3750-y

**Published:** 2019-10-21

**Authors:** Lucas Blattner, Reinhard Gerecke, Stefanie von Fumetti

**Affiliations:** 1 Department of Environmental Sciences, Geoecology Research Group, University of Basel, St. Johanns-Vorstadt 10, 4056 Basel, Switzerland; 2 Department of Biology, University of Tübingen, Auf der Morgenstelle 28E, 72076 Tübingen, Germany

**Keywords:** *cox*1, Barcoding, Species delimitation, Crenobiosis, *28S*, Springs, Biodiversity, Phylogeny, ABGD

## Abstract

**Background:**

Water mites are among the most diverse organisms inhabiting freshwater habitats and are considered as substantial part of the species communities in springs. As parasites, Hydrachnidia influence other invertebrates and play an important role in aquatic ecosystems. In Europe, 137 species are known to appear solely in or near springheads. New species are described frequently, especially with the help of molecular species identification and delimitation methods. The aim of this study was to verify the mainly morphology-based taxonomic knowledge of spring-inhabiting water mites of central Europe and to build a genetic species identification library.

**Methods:**

We sampled 65 crenobiontic species across the central Alps and tested the suitability of mitochondrial (*cox*1) and nuclear (*28S*) markers for species delimitation and identification purposes. To investigate both markers, distance- and phylogeny-based approaches were applied. The presence of a barcoding gap was tested by using the automated barcoding gap discovery tool and intra- and interspecific genetic distances were investigated. Furthermore, we analyzed phylogenetic relationships between different taxonomic levels.

**Results:**

A high degree of hidden diversity was observed. Seven taxa, morphologically identified as *Bandakia concreta* Thor, 1913, *Hygrobates norvegicus* (Thor, 1897), *Ljania bipapillata* Thor, 1898, *Partnunia steinmanni* Walter, 1906, *Wandesia racovitzai* Gledhill, 1970, *Wandesia thori* Schechtel, 1912 and *Zschokkea oblonga* Koenike, 1892, showed high intraspecific *cox*1 distances and each consisted of more than one phylogenetic clade. A clear intraspecific threshold between 5.6–6.0% K2P distance is suitable for species identification purposes. The monophyly of Hydrachnidia and the main superfamilies is evident with different species clearly separated into distinct clades. *cox*1 separates water mite species but is unsuitable for resolving higher taxonomic levels.

**Conclusions:**

Water mite species richness in springs is higher than has been suggested based on morphological species identification alone and further research is needed to evaluate the true diversity. The standard molecular species identification marker *cox*1 can be used to identify species but should be complemented by a nuclear marker, e.g. *28S*, to resolve taxonomic relationships. Our results contribute to the taxonomical knowledge on spring inhabiting Hydrachnida, which is indispensable for the development and implementation of modern environment assessment methods, e.g. metabarcoding, in spring ecology.
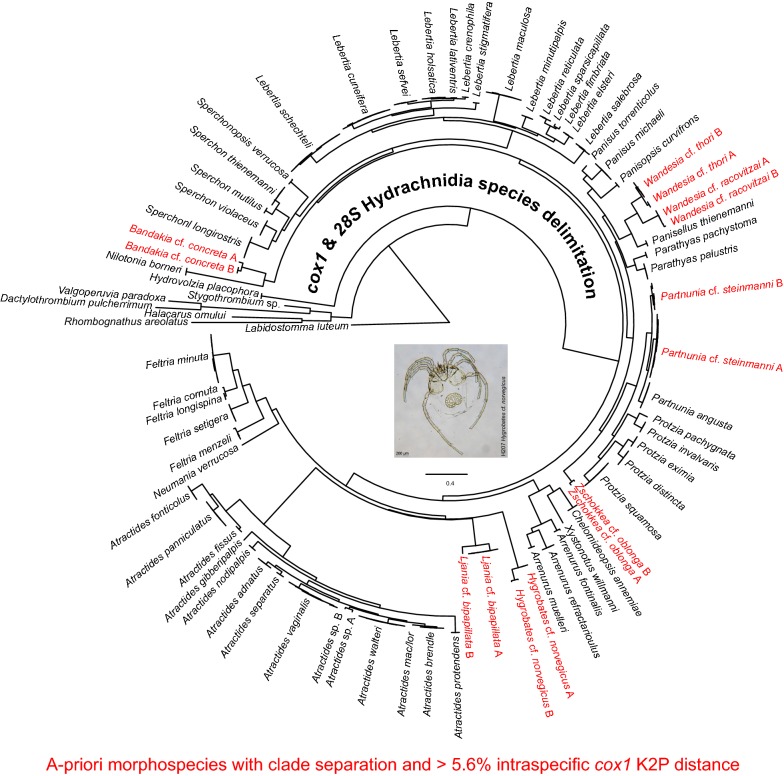

## Background

Water mites (Hydrachnidia) are highly diverse in aquatic habitats [1]. They have a complex life-cycle that includes a prelarva, a parasitic larval stage, an initial resting stage (protonymph), a free living deutonymph, a second resting stage (tritonymph) and the final adult stage [2]. Hydrachnidia disperse predominantly through passive rather than active pathways because water mite larvae parasitize other invertebrate taxa, generally insect hosts that fly [2–5].

Nearly all freshwater environments are inhabited by water mite species with a high degree of habitat specialization [6]. Mites in springs and other groundwater-influenced ecosystems occur in remarkably high diversity of habitats [7–9]. Of the 970 recorded European water mite species, 137 are found solely in or near springs and are adapted to several microhabitats, such as different substrate types and environmental conditions [2, 9]. Due to the high degree of adaptation and their influence on ecosystem functioning for other invertebrate taxa [10–14], these so called crenobiontic (occur exclusively in spring habitats) and crenophilous (tendency to be found in the spring brook) species play a critical role in spring species communities. Considering that springs are island-like habitats within an uninhabitable terrestrial matrix [15, 16], spring dwelling water mite populations are assumed to be rather isolated. This would promote reproductive isolation and therefore lead to an increased speciation rate [4, 17], which is among other things an explanation for the relatively high species diversity of water mites in springs. However, the degree of isolation of spring water mite populations is highly dependent on the dispersal abilities of their hosts and influenced by taxon specific host spectra and specificity [5, 18, 19]. Furthermore, the high microhabitat diversity in springs [15, 20], their relatively stable environmental conditions [21, 22] and absence of large predators, e.g. fish [9], make them exceptionally favorable habitats for insect larvae and benefit their development. Therefore, the diversity and abundance of water mite hosts is relatively high in springs compared to other freshwater habitats, which is likewise considered as precondition for the high number of crenobiont water mite species [9].

Despite their importance for freshwater species communities, the taxonomic knowledge about Hydrachnidia species is still limited today and new species are discovered frequently (e.g. [23–26]). The intensity of re-examination of European Hydrachnidia has increased over the past years with several taxonomic revisions published [9]. Most water mite species known today have been described based on morphology only and studies applying genetic methods to verify and complement these descriptions are still relatively scarce (e.g. [27–30]). Nonetheless, many studies have shown that genetic species delimitation, frequently using *cox*1 barcoding [31], has a large potential to reveal new species, resolve taxonomic questions and contribute to biodiversity baselines and assessments (e.g. [29, 32–35]). Species identifications and their ecological interactions are crucial for contributions to crenobiology, community ecology, developing reliable bioindicators and understanding population dynamics. Moreover, newly developed methods to monitor invertebrate assemblages in freshwater environments, such as the simultaneous identification of bulk sampled individuals (metabarcoding) [36] or the indirect community reconstruction by analyzing environmental DNA (eDNA) [37], rely on previously established genetic species reference databases. Thus, a proper taxonomical knowledge and species description is greatly needed.

Several factors are important to account for when using genetic species identification methods [38], such as the presence of endosymbionts like the alpha-proteobacteria *Wolbachia* sp. [39, 40] or the presence of pseudogenes and nuclear copies of mitochondrial DNA (numts) [41–43], which compromise the suitability of mitochondrial molecular markers to identify species. Standard barcoding methods are mainly based on sequence similarity and the relation between intra- and interspecific genetic distance, which is commonly calculated by using the Kimura 2-parameter (K2P) [44] and uncorrected (p) distances [31, 45, 46]. Nonetheless, the usability of genetic markers to identify species can vary between different taxonomic groups, geographical origin and sampling strategy [31, 38, 47], which implies a taxon-specific evaluation prior to a broad-scale application in environmental assessment and conservation.

In this study we use an integrative taxonomy approach to verify the species status and validity of the most common spring related Hydrachnidia species in Europe. We tested the reliability of techniques commonly used to identify and delimit species using fragments of the mitochondrial cytochrome *c* oxidase subunit 1 gene (*cox*1) [31, 48] and the D1-D2 region of the *LSU* rDNA gene (*28S*) [49], or using both (e.g. [28, 50–52]). This study aims at improving knowledge and analytical techniques for assessing Hydrachnidia diversity in springs and explores the strength and weaknesses of standardized barcoding loci to identify water mite species.

## Methods

### Water mite sampling

The studied crenobiontic and crenophilous Hydrachnidia species were sampled between 2008 and 2018 in 87 different sampling sites across Europe during multiple sampling occasions (Additional file 1: Table S1). Most of the specimens were collected from springs located within the protected areas Berchtesgaden National Park (Germany), Black Forest National Park (Germany), Gesäuse National Park (Austria) and in the Swiss National Park (Switzerland). Samples were manually collected with a 200-µm hand net. Water mites were either sampled alive in the field or sorted out in the laboratory under a stereomicroscope from mixed samples containing bulk substrate. All specimens were subsequently stored in ethanol (100%) and kept at 4 °C until further processing.

### Non-destructive DNA extraction

Total genomic DNA (gDNA) of each individual mite was extracted by using either GeneReleaser® (BioVentures, Murfreesboro, TN, USA) or the DNeasy Blood & Tissue Kit (Qiagen, Hilden, Germany). Both methods allow a non-destructive DNA extraction, which is essential when voucher specimens need to be retained for morphological identification in barcoding projects. Prior to both extraction methods, each individual was cleaned by using forceps and entomological needles in a small Petri dish filled with ethanol (100%) under a stereomicroscope. All instruments and vessels used were cleaned after processing each mite specimen by rinsing it with sodium hypochlorite (13%), molecular grade water and ethanol (80%). Afterwards, the specimens were air dried and soaked in molecular grade water for 3 min to ensure the absence of ethanol residues. The cleaned specimen was then transferred to either a 0.2-ml PCR tube containing 0.9 µl of molecular grade water and 0.1 µl of 1× PCR buffer (Qiagen) in the case of GeneReleaser^®^ or to a 1.5-ml tube containing 180 µl of buffer ATL (Qiagen) and 20 µl (20 mg/ml) of Proteinase K (Qiagen) when using the DNeasy Blood & Tissue Kit. The GeneReleaser^®^ method was conducted as originally described by Schizas et al. [53] and modified by Böttger-Schnack & Machida [54], see also [55]. Instead of resuspending the supernatant in TE buffer, step 6 of the modified protocol [54], approximately 12 µl of supernatant was transferred into a new 0.2-ml PCR tube and used directly as DNA template for the subsequent PCR reactions. The DNeasy Blood & Tissue extraction was performed according to the manufacturer’s protocol (Animal Tissues, Spin-Column Protocol, Qiagen) with minor changes. The specimens were incubated in buffer ATL and Proteinase K at 56 °C on a shaking thermomixer (400× *rpm*) overnight (step 2 in the manufacturer’s protocol) and the elution buffer (AE) volume was decreased to 100 µl in the last step to increase the gDNA concentration. The concentration of every DNA template was measured after the extraction by using a Qbit 3.0 Fluorometer (Thermo Fisher Scientific, Waltham, MA, USA) and the dsDNA HS Assay Kit (Thermo Fisher Scientific). After the first processed specimens it was evident that the mean amount of total gDNA obtained by the DNeasy procedure is higher (mean ± SD: 71.5 ± 2.3 ng in 100 µl of solution, *n* = 92) than when extracting gDNA by using the GeneReleaser^®^ method (mean ± SD: 56.8 ± 4.8 ng in 12 µl of solution, *n* = 105). Therefore, the DNeasy method was chosen for all subsequent extractions.

### Morphological examination

All water mite individuals were identified morphologically by the authors RG and LB using current Hydrachnidia identification keys [56–58]. After the DNA extraction, the mite specimens were dissected and mounted on slides in Hoyer’s medium or identified as whole individuals under a compound microscope when possible. The enzymatic DNA extraction method (Proteinase K) leads to a partial digestion of the specimens causing discoloration and therefore improved visibility of morphological characters, especially of sclerotized parts. This often allows the morphological identification without dissection. However, digestion is a process that affects membranous parts and therefore deteriorates the observability of integument structures such as the papillae, tubercles or lining. All voucher specimens are stored in the acarological collection of the Natural History Museum Basel (Switzerland) under the museum identifications presented in Additional file 1: Table S1.

### PCR amplification and sequencing

The approximate 650 bp standard barcoding fragment of the cytochrome *c* oxidase subunit 1 (*cox*1) [31] mitochondrial gene was first amplified by using universal primers LCO1490 and HCO2198 [59] of a subset of Hydrachnidia species belonging to several genera (*Atractides*, *Feltria*, *Hygrobates*, *Lebertia*, *Partnunia*, *Protzia* and *Sperchon*). PCR reactions contained 0.25 µl of Phusion High Fidelity DNA Polymerase (2 U/µl) (Thermo Fisher Scientific), 5 µl of 5× Phusion HF Buffer (Thermo Fisher Scientific), 0.5 µl of dNTP mix (10 mM) (Sigma-Aldrich, Buchs, SG, Switzerland), 1.25 µl of forward and reverse primers (10 µM each), 5 µl of template DNA and ultrapure water to a total reaction volume of 25 µl. The PCR conditions were as follows: initial denaturation for 30 s at 98 °C; 35 cycles of 10 s at 98 °C, 30 s at 50 °C and 30 s at 72 °C; final elongation for 2 min at 72 °C. PCR products were then stored at 4 °C. As this primer combination failed to amplify several samples we decided to design new genus-specific *cox*1 barcoding primers on the basis of the previously amplified water mite specimens. This was possible due to a low sequence variability at the 5’ and 3’ ends of the barcoding fragment.

Additionally, a new set of universal barcoding primers was designed by degenerating and modifying several positions of the original LCO1490/HCO2198 primers to enable a higher amplification performance when barcoding Hydrachnidia species. These new primer sets (Table 1) were used to amplify all remaining samples. The subsequent PCRs were performed by using 0.25 of µl Q5^®^ High-Fidelity DNA Polymerase (2U/µl) (NEB, Ipswich, USA), 5 µl of 5× Q5^®^ Reaction Buffer (NEB), 0.5 µl of dNTP mix (10 mM) (Sigma-Aldrich), 1.25 µl of forward and reverse primers (10 µM each), 5 µl of template DNA and ultrapure water to a total reaction volume of 25 µl. The PCR conditions were the same for all newly designed *cox*1 primer sets and were as follows: initial denaturation for 30 s at 98 °C; 35 cycles of 10 s at 98 °C, 30 s at 51 °C and 20 s at 72 °C; and a final elongation step for 2 min at 72 °C. The PCR products were then kept at 4 °C until further processing. To amplify the D1-D2 domain of the *LSU* rRNA *28S* gene we designed new water mite-specific primer sets on the basis of the D1D2fw2 forward primer [49] and by aligning different *28S* Hydrachnidia sequences downloaded from GenBank. The new primers 28SHy_F and 28SHy_R (Table 1) reliably amplified an approximately 1.2 kbp long fragment of the *28S* D1-D2 domain. The *28S* PCR reactions were done by using the same reaction components and conditions as used when amplifying with the new *cox*1 primer sets. The only difference was a higher annealing temperature at 68 °C instead of 51 °C. All PCR primers (*cox*1 and *28S*) were tailed with modified M13 sequences (M13: 5′-TGT AAA ACG ACG GCC AG-3′ and M13r: 5′-CAG GAA ACA GCT ATG AC-3′) [60], which has shown to improve the amplification and sequencing reactions in this and previous studies [61, 62]. Prior to sequencing, PCR products where examined on an agarose gel electrophoresis and purified using the QIAquick PCR Purification Kit (Qiagen) according to the manufacturer’s protocol on a QIAcube (Qiagen). The purified PCR products were Sanger sequenced with the above mentioned M13 primers by Mycrosynth AG (Balgach, Switzerland).

**Table 1:** Primers designed and used in this study

Marker	Taxon	Name	Direction	Sequence (5′–3′)
*cox*1	Hydrachnidia	LCO_Hydr	F	CAACAAACCAYAAAGAYATTGG
		HCO_Hydr	R	TGGGTGTCCRAARAATCA
	*Atractides*	Atr_F	F	ACCAYAAAGAYATTGGAAC
		Atr_R	R	AAAATCAGAARATATGTTGA
	*Lebertia*	Leb_F	F	CAAACCAYAAAGAYATTGGAAC
		Leb_R	R	CGAAGAATCAAAATARRTGTTG
	*Partnunia*	Part_F	F	ACACTYTACTTYGCTTTTGG
		Part_R	R	CAAAGAATCAAAATAARTGTTG
	*Feltria*	Fe_F	F	ATATTGGYACTTTATATTTCGG
		Fe_R	R	CGAAGAATCAAAATARATGTTG
	*Protzia*	Leb_F	F	CAAACCAYAAAGAYATTGGAAC
		Protz_R	R	GATGTRTTAAARTTTCGATCTG
	*Hydrovolzia*	Hydrov_F	F	TGGGCWGGAATTTTAGGATC
		Hydrov_R	R	TGTTGAAAGAGGATTGGGTC
	*Hygrobates*	Riv_F	F	CAAACCAYAAAGAYATTGGTAC
		HCO_Hydr	R	TGGGTGTCCRAARAATCA
	*Wandesia*	Wand_F	F	ACCAYAAAGAYATTGGGACC
		HCO_Hydr	R	TGGGTGTCCRAARAATCA
*28S*	Hydrachnidia	28SHy_F	F	AGTACCGTGAGGGAAAGTTG
		28SHy_R	R	GGCAGGTGAGTTGTTACACA

*Abbreviations*: F, forward; R, reverse

### Molecular analysis

Raw sequences were analyzed, edited and aligned in Geneious Prime v.2019.1.1 [63]. Low-quality base calls, ambiguous sites and primer binding sites at the 5′- and 3′-ends were trimmed prior to further processing. Alignments were done by using MAFFT v.7.388 [64] implemented in Geneious Prime. Potentially poorly aligned positions and divergent regions of the alignments were eliminated with Gblocks v.0.91b [65, 66]. All sequences were tested for the presence of contaminants by blasting with the Nucleotide Blast Tool (BLASTn) implemented on the NCBI website [67]. Because misleading numts can be amplified in PCRs targeting *cox*1 mtDNA, we translated the sequences into amino acids to check for the presence of stop codons, which is commonly seen as a suitable way to detect erroneous amplification [41]. The concatenated alignment containing *cox*1 and *28S* sequences was generated by Sequence Matrix v.1.8 [68]. The suitable nucleotide substitution model for each marker (*cox1*: TPM2uf+I+G4 and *28S*: TVM+I+G4) was selected according to the Bayesian information criterion (BIC) as implemented in ModelTest-NG v.0.1.5 [69], a novel software, which combines features of jModelTest2 [70] and ProtTest3 [71] on the CIPRES Science Gateway v.3.3 [72]. All sequences generated in this study are deposited in NCBI GenBank under the accession numbers MK889511–MK889751 (*cox*1) and MK889752–MK889992 (*28S*) and on BOLDsystems under the IDs LBCWS001-19 to LBCWS245-19 (*cox*1).

### Distance-based species delimitation

Intra- and interspecific Kimura 2-parameter (K2P) [44] and uncorrected (p) distances were calculated in MEGA X [73]. The species delimitation threshold was investigated by using the threshold optimization method of the SPIDER (Species Identity and Evolution in R) v.1.5.0 package [74] implemented in R [75] as described in the tutorial (available at: http://spider.r-forge.r-project.org). Additionally, we used the Automated Barcode Gap Discovery (ABGD) procedure [76] to assign the sequences to hypothetical species based on the gap between intra- and interspecific sequence diversity, the so-called “barcoding gap”. ABGD was performed on the ABGD web interface [77] by using the MEGA distance files with default parameters, 20 steps and a modified relative gap width of 1. Additionally, the sequences were analyzed by using the Bold Systems v.4 [78, 79] tools available on the Barcode of Life webpage [80].

### Phylogenetic species delimitation

Phylogenetic relationships between the sampled Hydrachnidia species were examined with maximum likelihood (ML) and Bayesian inference (BI). RAxML-NG [81], which is a new improved version of RAxML [82], was used to infer the best fitting ML trees of the single markers (*28S* and *cox*1) and the concatenated alignment, respectively. Similar sequences were treated as duplicates and removed automatically by RAxML-NG at the beginning of the tree calculation. ML branch support values were generated by the bootstrap method [83] with 1000 replicates and bipartition support for the best ML tree. Bootstrapping trees were computed directly in RAxML-NG. The BI trees were generated by using the parallel MPI version of MrBayes v.3.2.6 [84, 85]. Bayesian inferences were run for 15 × 10^6^ MCMC generations, sampled every 5000th generation after the exclusion of 25% ‘burn-in’ by using 4 independent chains. Branches showing bootstrap values below 70% and Bayesian posterior probabilities below 0.95 were interpreted as resolved but not statistically supported [86]. The concatenated alignment was treated as partitioned dataset with unlinked base frequencies, nucleotide substitution rates, gamma shapes and proportions of invariant sites. The rates and frequencies were set according to the ModelTest-NG results. For each marker (*cox*1 and *28S*), the appropriate nucleotide substitution model was used when running RAxML-NG and MrBayes for the single and partitioned analysis, respectively. To resolve the basal nodes and ensure a reliable rooting, several outgroup taxa were added to the tree inferences. The most distant taxon included was the terrestrial mite *Labidostomma luteum* Kramer, 1879 (Labidostommatoidea) (GenBank *28S*/*cox*1: KM100974/GQ864390). Additionally, *Dactylothrombium pulcherrimum* (Haller, 1882) (Trombidioidea) (GenBank: KM100939/KM100985), *Valgoperuvia paradoxa* (Robaux, 1970) (Trombidioidea) (GenBank: KM100943/KM100988) and *Stygothrombium* sp. (Stygotrombidioidea) (GenBank: KM100938/ ﻿KM100995) sequences were used as closely related terrestrial Acariformes. The Halacaridae species *Halacarus omului* (Pepato & Da Silveira, 2013) (GenBank: MG751425/MG696236) and *Rhombognathus areolatus* (Abé & Fernandes, 2011) (GenBank: MG751437/MG696244) were chosen as aquatic relatives to the monophyletic Hydrachnidia [87]. The final trees were analyzed and edited in FigTree v.1.4.4 [88], Geneious Prime v.2019.1.1, Dendroscope v3.5.10 [89] and Affinity Designer v.1.6.1 (Serif Europe Ltd., Nottingham, UK).

## Results

We successfully amplified and sequenced both target loci (*cox*1 and *28S*, respectively) of 241 individual water mite specimens representing 22 genera and 65 morphologically identified crenobiontic and crenophilous species with 1 to 19 individuals per species (Additional file 1: Table S1). Three individuals belonging to the genus *Atractides* sp. (H450, H528 and H531), all representatives of the *loricatus* species group, were not identifiable to species level. As also observed in other populations of this group collected in various parts of Europe, important diagnostic features, i.e. large *vs* small dorsal muscle attachment sclerites, size of acetabula in the genital field and sclerotized or smooth excretory pore, as well as character state combinations are in disagreement with the identification key in Gerecke et al. [58].

*cox*1 final alignment length was 650 bp, 398 sites were polymorphic (389 parsimony informative) and no alignment gaps were present. The 999 bp *28S* alignment showed 466 polymorphic sites (358 parsimony informative) and 137 gap positions. The translation of the *cox*1 sequences into amino acids did not contain any stop codon positions and blasting the sequences confirmed the absence of contaminations. In a few cases, when using the universal primer pair (LCO1490/HCO2198), we amplified Chironomidae DNA instead of water mite DNA and discovered *Wolbachia* sp. infestation. These specimens were excluded from further analysis.

### Distance-based species delimitation and discovery

**Fig. 1:**
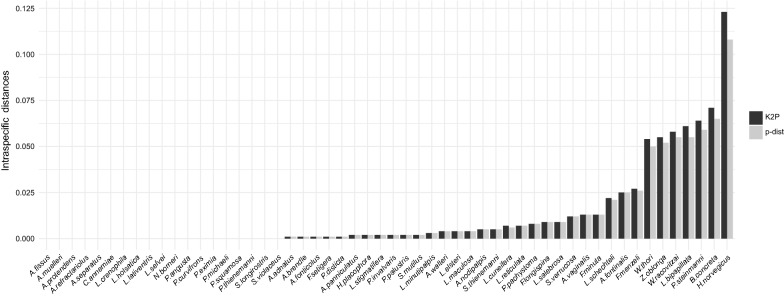
Intraspecific *cox*1 distances. K2P and p-distances within morphologically identified species represented by more than one individual

The mean overall pairwise distances were larger between the *cox*1 sequences (K2P ± SD: 0.29 ± 0.10; p-distance ± SD: 0.24 ± 0.07) compared to *28S* (K2P ± SD: 0.15 ± 0.10; p-distance ± SD: 0.14 ± 0.05). Out of the 65 morphologically identified taxa, 11 were singletons, 7 taxa (*Bandakia concreta* Thor, 1913, *Hygrobates norvegicus* (Thor, 1897), *Ljania bipapillata* Thor, 1898, *Partnunia steinmanni* Walter, 1906, *Wandesia racovitzai* Gledhill, 1970, *Wandesia thori* Schechtel, 1912 and *Zschokkea oblonga* Koenike, 1892) showed exceptionally high intraspecific *cox*1 K2P distances (> 0.05) and the majority (47 species) had within species K2P distances between 0 and 0.03 (Fig. 1). *Hygrobates norvegicus* exhibited the largest mean genetic *cox*1 distances within morphologically identified species (K2P ± SD: 0.12 ± 0.08; p-distance ± SD: 0.10 ± 0.07), whereas several species showed mean intraspecific K2P distanced below 0.01 (< 1%) (Fig. 1).

The SPIDER threshold optimization procedure analysis, which was conducted after the removal of singleton species and sequences of the seven taxa showing exceptionally high intraspecific variation, revealed an optimal K2P distance threshold at 0.056 (5.6%) and a p-distance threshold at 0.053 (5.3%) for species identification purposes with no false positive and low false negative identifications (9 out of 225 sequences). Assuming that *cox*1 species identification threshold, the individuals belonging to the above-mentioned taxa, which show high intraspecific variation, are likely to represent more than one species. This threshold was confirmed by the ABGD method that indicated a barcoding gap between K2P and p-distances of 0.06 and 0.09 (Additional file 2: Figure S1). ABGD initial partition revealed 69 and the recursive partition 70 groups, which can be seen as equivalent to species. Each of the seven taxa showing high intraspecific distances were split into two separate groups. Additionally, *Lebertia schechteli* Thor, 1913 showed clade separation in the recursive but not the initial partition causing the disparate number of groups between the partitions (Additional files 3 and 4: Figures S2 and S3). This generally confirms the SPIDER results and indicates the presence of more species than *the a priori* identified morphospecies. Contrary to that, *Lebertia crenophila* Viets, 1920, *Lebertia holsatica* Viets, 1920 and *Lebertia lativentris* Viets, 1922 as well as *Atractides macrolaminatus/A. loricatus* and *Atractides brendle* Gerecke, 2003 were grouped together as the same species in both ABGD partitions. The same analyses were conducted for the *28S* dataset. However, neither the ABGD nor the SPIDER method revealed a threshold suitable for species identification purposes. ABGD indicated the absence of a clear barcoding gap for the *28S* locus (Additional file 5: Figure S4) and the threshold optimization implemented in the SPIDER R package indicated high levels of false negative and false positive identifications at different thresholds.

### Phylogenetic species delimitation and discovery

**Fig. 2:**
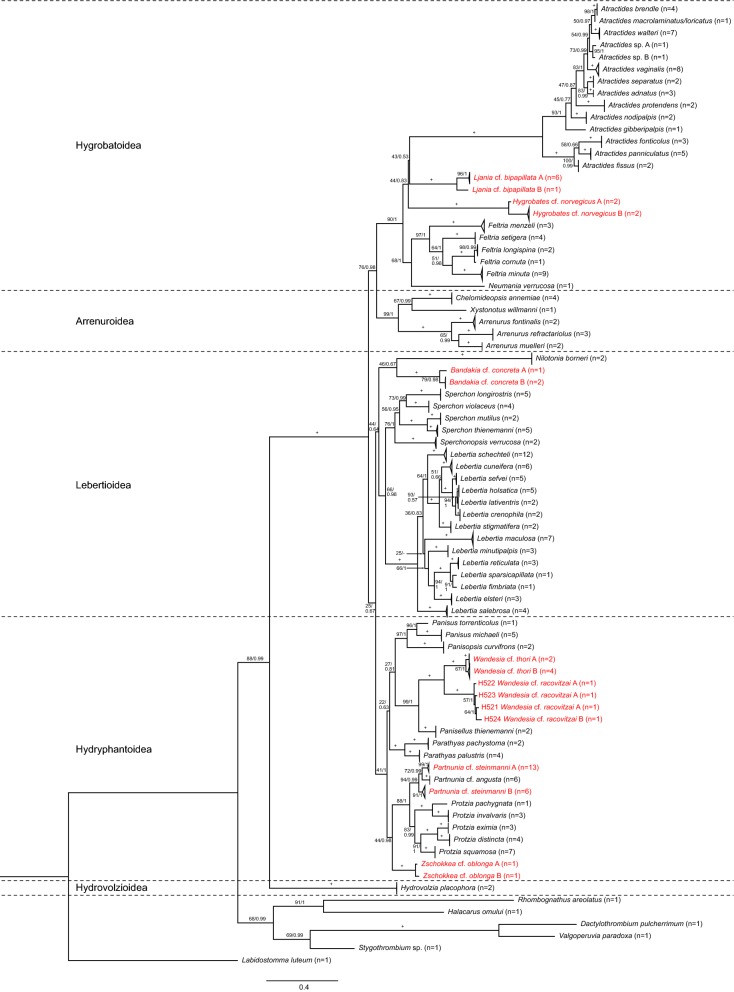
Maximum likelihood tree of the combined *cox*1 and *28S* datasets. Support values are shown as bootstrap (BS) and posterior probability (PP) values (PP/BS). Nodes fully supported by PP* = *1 and BS* = *100 are shown as +. In the case of clear monophyletic clades, tip nodes with more than one individual were collapsed with the number of individuals indicated as (*n = *X). In the case of clade separation within morphologically identified species we named the corresponding specimens by using the species name, cf. and A & B. Clades containing more molecular species than the *a priori* morphospecies are marked in red. Specimen IDs and sampling data of the individuals belonging to species are provided in Additional file 1: Table S1

The results obtained by the distance-based species delimitations were generally confirmed by the phylogenetic approach. However, in contrast to the combined *28S* and *cox*1 analysis, the single marker datasets alone did not allow to correctly reveal phylogenetic relationships at different taxonomic ranks and the BI trees (Additional files 6 and 7: Trees S1 and S2) showed several unresolved nodes and polytomies compared to the better resolved ML trees (Additional files 8 and 9: Figures S5 and S6). *cox*1 analyses incorrectly clustered higher taxonomic levels (e.g. genus, family and superfamily). For example, the genus *Protzia* Piersing, 1896 was clustered together with *Sperchon* Kramer, 1877 instead of the more closely related *Partnunia* Piersing, 1896 (Additional file 8: Figure S5). *28S* correctly reconstructed higher taxonomic levels but did not allow resolving species relationships in several cases, e.g. *Feltria cornuta* Walter, 1927 and *Feltria longispina* Motas & C. Angelier, 1927 or *Lebertia holsatica* Viets, 1920 and *Lebertia lativentris* Viets, 1922 (Additional file 9: Figure S6). Compared to the single marker analysis, the overall taxonomic relatedness was depicted correctly by the combined dataset (Fig. 2). Furthermore, both phylogenetic methods (ML and BI) showed largely congruent and stable tree topologies when applied to the combined *cox*1 and *28S* alignment (Additional file 10: Alignment S1). Due to these findings, we will focus on the ML tree with combined branch support data (Fig. 2).

Results showed that the superfamilies (Hydrovolzioidea, Hydryphantoidea, Lebertioidea, Arrenuroidea and Hygrobatoidea) are monophyletic and clearly separated from each other. A relatively distinct clade affiliation of individuals belonging to the morphologically identified species is evident (Fig. 2). The previously mentioned specimens showing high intraspecific distances are also clearly separated into different clades. All corresponding branches showed high support values, indicating high probabilities of these splits. In the case of *Partnunia steinmanni*, individuals are grouped in two distinct clades, *Partnunia* cf. *steinmanni* A that shares a common ancestor with brook inhabiting (rhithrobiont) *Partnunia angusta* (Koenike, 1893) and *Partnunia* cf. *steinmanni* B. Both morphologically unidentifiable *Atractides* sp. (A & B) individuals represent two genetic species and also the individual belonging to the *Atractides* gr. *macrolaminatus/loricatus* is clearly separated from all other *Atractides* sp. specimens (Fig. 2).

## Discussion

Morphological species identification has a long tradition and is commonly used to identify species for scientific and applied (e.g. ecosystem assessment) purposes [90]. Recently, it has been shown that molecular data reliably complement morphological species identification and has many advantages, especially when used to identify multiple species at once [36, 91]. Furthermore, molecular species delimitation resolves taxonomic uncertainties. When combined with other species-defining characteristics such as morphology it produces a more complete conclusion (e.g. [34, 35, 92, 93]).

Our results generally confirm the morphological species delimitation but show that Hydrachnidia species richness is underestimated and molecular methods are essential to discover currently overlooked biodiversity. The distance-based species delimitation methods revealed an intraspecific *cox*1 threshold between 5.6% (SPIDER) and 6% (ABGD) K2P distance, which is relatively high compared to other taxa [31, 48] but seems to be typical in water mites [28, 29, 94]. However, species delimitation solely based on fixed genetic distance thresholds can be misleading and thresholds should be estimated individually for each dataset [38, 95, 96], especially in taxa with clade-specific intraspecific *cox*1 distances as demonstrated for water mites in this and previous studies (e.g. [94, 97]). As the *28S* marker region did not show a distinct barcoding gap and a clear species identification threshold was not evident, we do not recommend using it as single marker for threshold-based species identification of water mites.

The distance- and morphology-based results were confirmed by applying a phylogenetic approach. A clear monophyletic clade affiliation of individuals belonging to the same species was evident. Both, the distance-based and the phylogeny-based species delimitation revealed that seven morphologically identified species (*Bandakia concreta*, *Hygrobates norvegicus*, *Ljania bipapillata*, *Partnunia steinmanni*, *Wandesia racovitzai*, *Wandesia thori* and *Zschokkea oblonga*) show high genetic differences and therefore are likely to represent more than one species. The *cox*1 ABGD results differed in two cases from the other methods. *Lebertia crenophila*, *L. holsatica* and *L. lativentris* were grouped together as one species as it was also the case in *Atractides macrolaminatus/loricatus* and *A. brendle*. However, these species phylogenetically belong to different clades with high support values and we therefore assume that ABGD erroneously groups the respective sequences and underestimates the number of species, a tendency that has already been shown by other studies (e.g. [98]). Furthermore, ABGD initial and the recursive partition differently grouped *L. schechteli* individuals either as one single or two separate species. The initial partition is considered as more stable and usually better represents the groups that are defined by taxonomists [76, 99]. In addition, the phylogenetic inferences revealed a distinct *L. schechteli* clade. Therefore, we assume that our *L. schechteli* specimens belong to a single species. However, the recursive partition results show the possibility of differentiation and indicate that further research is advisable. In the analysis of *H. norvegicus* and *P. steinmanni* we were able to include several individuals per genotype, whereas in the other taxa differentiation is based on single individuals. Therefore, further individuals of different populations will be processed prior to the final description of the potentially new species.

Results suggest the monophyly of Hydrachnidia and the previously defined superfamilies, supporting the findings of Dabert et al. [87]. In contrast to the latter authors, and possibly due to the inclusion of more taxa in this study, our data support a Hydryphantoidea monophylum. However, the corresponding node separating Hydryphantoidea and Lebertioidea is poorly supported in our results and therefore should be further questioned. Similar to the study of Dabert et al. [87], we equally observed that *cox*1 or *28S* alone are incapable of fully resolving phylogenetic relationships. This phenomenon can probably be caused by mito-nuclear discordance, which has already been recorded for mites and other taxa [100, 101] and was reviewed intensively [102]. Besides this assumption postulating diverging nuclear and mitochondrial phylogenies, the different taxonomic resolution of the two marker regions is likely caused by the loss of phylogenetic information. This can be explained by the fast-evolving character of mitochondrial compared to nuclear DNA and therefore faster loss of ancestral polymorphisms in *cox*1 [102–105]. Arabi et al. [105] showed exceptionally high mitogenomic rearrangements especially in Chelicerata, which fosters this assumption. Therefore, we conclude the necessity of complementing the standard barcoding marker *cox*1 with at least one additional genetic marker, e.g. *28S* or *18S* rDNA, to investigate species relationships and fully resolve water mite taxonomy.

These findings show that the choice of species identification markers must be done with caution and should be adjusted to the research question. For Hydrachnidia, *cox*1 serves as a useful marker if solely species identification is of interest. If the goal is to assign sequences to higher taxonomic levels, another locus should be taken into account. This is also crucial if the aim is to assess the amount of undescribed species in an environment. Currently, most metabarcoding approaches are based on *cox*1 alone [106–109] and few studies investigated the performance of alternative loci (e.g. [110–112]). Apart from the taxon assignment limitations when using *cox*1 alone, our findings show that the primer bias problem [36, 111] needs to be considered when water mites are targeted in metabarcoding studies as universal *cox*1 primers show unsatisfactory amplification performance. This could potentially be circumvented by using a combination of more specific *cox*1 primer sets as has been done in this study, a better matching universal one or the establishment of a new metabarcoding marker for this taxon. We were able to show that molecular methods have a great potential to reveal new water mite species and more studies are needed to complete barcoding databases and refine biodiversity estimates. Fundamental knowledge of species diversity is an essential precondition for implementing water mites in recent monitoring approaches and use them as powerful bioindicators [113, 114] in freshwater assessments as for example required by the European Water Framework Directive (WFD) [115], and may also offer an opportunity for a more nuanced understanding of environmental change impacts on springs systems.

Looking at spring inhabiting Hydrachnidia species, our work contributes to the accumulation of species barcoding data. Our data covers 47.5% of the currently described 137 spring water mite species in Europe [9] and covers the most abundant taxa, especially in the central Alps. Compared to other studies, which investigate Hydrachnidia diversity by applying morphological and molecular techniques in other aquatic habitats than springs (e.g. [28, 87, 94, 116, 117]), we were able to include a large subset of different species belonging to different taxonomic groups including the Proto-, Eu- and Neohydrachnidia [87]. A relatively high proportion of morphologically identified species (10.8%) showed to be more diversified than had been assumed, which indicates an overall underestimation of Hydrachnidia species richness in springs and other aquatic habitats. This indicates that species diversity related research questions such as host specificity of different water mite species need to be reconsidered. *Hygrobates norvegicus*, *Partnunia steinmanni* and *Ljania bipapillata* were shown to have a rather wide host species spectrum [18, 19]. Considering our results, which indicate that these three morphologically described taxa consist of several novel species, the number of hosts per water mite species could be lower and reveal a tendency towards high host specificity.

The dispersal abilities of Hydrachnidia highly depend on the parasitic larval stage that attaches to an insect host that can carry it to a different habitat and therefore governs water mite presence or absence in an environment [2, 13, 118]. Especially in rather isolated freshwater habitats like springs, water mite species dispersal is directly linked with their specific hosts leading to the conclusion that phylogeographic patterns are shared between hosts and parasites. Combined with our assumption of increased host specificity due to the unexpected high degree of Hydrachnidia species diversification, future studies on gene flow patterns between water mite populations can verify the hypothesis that springs are isolated island-like habitats for the mites as well as their insect hosts.

In Europe, 970 water mite species have been recorded to date [9] and, applying our findings, at least 105 additional species potentially exist. Due to the fact that water mite species diversity increases towards southern parts of Europe [9] we expect an even higher degree of undiscovered species as our dataset mainly consists of specimens collected in central Europe. On this basis, future water mite barcoding projects will be able to gradually fill the gaps of taxonomic knowledge. This is an important prerequisite to incorporating modern species identification and monitoring techniques (e.g. metabarcoding) in future water mite-related freshwater and spring assessment studies.

## Conclusions

Our aim was to verify and complement the mainly morphology-based species delimitation of an often neglected, highly diverse taxon in freshwater ecosystems. Our results show that water mite diversity in springs seems to be higher than expected. Molecular methods are largely congruent with morphology and serve as a species delimitation and identification tool. They are particularly powerful if species discovery is the main goal. *cox*1 as a standard barcoding marker is useful for identifying Hydrachnidia species but is not suitable for assigning them to higher taxonomic levels (e.g. genera, families or superfamilies). This limitation can be overcome by using distance- and phylogeny-based multi marker approaches. Our data contributes to genetic species identification databases by adding crenobiontic water mite sequences, which is a precondition for implementing modern methods of freshwater ecosystem assessment such as metabarcoding and eDNA species community monitoring in spring ecology.

## Supplementary information

**Additional file 1: Table S1.** List of specimens with sampling data, voucher museum IDs (Natural History Museum of Basel) and the sequence accession numbers.

**Additional file 2: Figure S1.** ABGD output plots of the *cox*1 K2P (A&B) and p-distances (C&D).

**Additional file 3: Figure S2.** Initial partition ABGD *cox*1 output tree. Shows individuals grouped as putative species delimited by the ABGD method. Clades indicating more species than the a-priori morphospecies are marked in red.

**Additional file 4: Figure S3.** Recursive partition ABGD *cox*1 output tree. Shows individuals grouped as putative species delimited by the ABGD method. Clades indicating more species than the a-priori morphospecies are marked in red and differences to the initial partition in blue.

**Additional file 5: Figure S4.** ABGD output plots of the *28S* K2P (A&B) and p-distances (C&D).

**Additional file 6: Tree S1.** *cox*1 phylogenetic BI tree MrBayes output file containing support values (posterior probability) (can be viewed in the program FigTree).

**Additional file 7: Tree S2.** *28S* phylogenetic BI tree MrBayes output file containing support values (posterior probability) (can be viewed in the program FigTree).

**Additional file 8: Figure S5.** *cox*1 RAxML-NG Maximum Likelihood tree with support values. Clades indicating more species than the a-priori morphospecies are marked in red.

**Additional file 9: Figure S6.** *28S* RAxML-NG Maximum Likelihood tree with support values. Clades indicating more species than the a-priori morphospecies are marked in red.

**Additional file 10: Alignment S1.** MAFFT Alignment of the combined (*cox*1 and *28S*) dataset and the root GenBank sequences, used to generate the phylogenetic tree.

## Data Availability

Data analyzed during this study are included in this published article and its additional files. New sequences generated in this work were deposited in the GenBank database under the accession numbers MK889511–MK889751 (*cox*1) and MK889752–MK889992 (*28S*) and on BOLDsystems under the IDs LBCWS001-19 to LBCWS245-19 (*cox*1). Water mite voucher specimens are stored in the acarological collection of the Natural History Museum Basel (Switzerland) under the identifications NMB-TROM-10000 to NMB-TROM-10240.

## Abbreviations

*28S*: large subunit ribosomal RNA gene
*18S*: small subunit ribosomal RNA gene
ABGD: Automatic Barcode Gap Discovery (species delimitation algorithm)
BI: Bayesian inference (phylogenetic tree inference method)
BIC: Bayesian information criterion
BLASTn: Nucleotide Basic Local Alignment Search Tool
BOLD: Barcode of Life Database
BS: bootstrap
*cox*1: cytochrome *c* oxidase subunit 1 gene
eDNA: environmental DNA
G: Gamma parameter
gDNA: genomic DNA
I: invariant sites
K2P: Kimura 2-parameter
LSU: large subunit
MAFFT: multiple alignment using fast Fourier transform (sequence alignment algorithm)
MCMC: Markov chain Monte Carlo
MEGA: Molecular Evolutionary Genetic Analysis (molecular genetics software)
ML: maximum likelihood (phylogenetic tree inference method)
MPI: message passing interface
numts: nuclear copies of mitochondrial DNA
PP: posterior probability
RAxML: randomized accelerated maximum likelihood (phylogenetic tree inference software)
rDNA: ribosomal DNA
SPIDER: Species Identity and Evolution in R
WFD: Water Framework Directive

## References

[1] Di Sabatino A, Smit H, Gerecke R, Goldschmidt T, Matsumoto N, Cicolani B (2008). Global diversity of water mites (Acari, Hydrachnidia; Arachnida) in freshwater. Hydrobiologia..

[2] Di Sabatino A, Gerecke R, Martin P (2000). The biology and ecology of lotic water mites (Hydrachnidia). Freshw Biol..

[3] Bohonak AJ (1999). Effect of insect-mediated dispersal on the genetic structure of postglacial water mite populations. Heredity..

[4] Bilton DT, Freeland JR, Okamura B (2001). Dispersal in freshwater invertebrates. Annu Rev Ecol Syst..

[5] Zawal A (2003). The role of insects in the dispersion of water mites. Acta Biol Univ Daugavp..

[6] Walter DE, Proctor HC (2013). Mites: ecology, evolution & behaviour.

[7] Stoch F, Gerecke R, Pieri V, Rossetti G, Sambugar B (2011). Exploring species distribution of spring meiofauna (Annelida, Acari, Crustacea) in the south-eastern Alps. J Limnol..

[8] Di Sabatino A, Cicolani B, Gerecke R (2003). Biodiversity and distribution of water mites (Acari, Hydrachnidia) in spring habitats. Freshw Biol..

[9] Gerecke R, Martin P, Gledhill T (2018). Water mites (Acari: Parasitengona: Hydrachnidia) as inhabitants of groundwater-influenced habitats - considerations following an update of Limnofauna Europaea. Limnologica..

[10] Lanciani C (1979). The influence of parasitic water mites on the instantaneous death rate of their hosts. Oecologia..

[11] Smith BP (1988). Host-parasite interaction and impact of larval water mites on insects. Ann Rev Entomol..

[12] Smith IM (1991). Water mites (Acari: Parasitengona: Hydrachnidia) of spring habitats in Canada. Mem Entomol Soc Canada..

[13] Martin P, Gerecke R (2009). Diptera as hosts of water mite larvae—an interesting relationship with many open questions tionship with many open questions. Lauterbornia..

[14] Werblow A, Martin P, Dörge DD, Koch LK, Mehlhorn H, Melaun C (2015). Hyperparasitism of mosquitoes by water mite larvae. Parasitol Res..

[15] Cantonati M, Gerecke R, Bertuzzi E (2006). Springs of the Alps—sensitive ecosystems to environmental change: from biodiversity assessments to long-term studies. Hydrobiologia..

[16] Von Fumetti S, Blattner L (2016). Faunistic assemblages of natural springs in different areas in the Swiss National Park: a small-scale comparison. Hydrobiologia..

[17] Rabosky DL (2016). Reproductive isolation and the causes of speciation rate variation in nature. Biol J Linn Soc..

[18] Martin P, Stur E, Wiedenbrug S (2009). Larval parasitism of spring-dwelling alpine water mites (Hydrachnidia, Acari): a study with particular reference to chironomid hosts. Aquat Ecol..

[19] Martin P, Stur E (2006). Parasite-host associations and life cycles of spring-living water mites (Hydrachnidia, Acari) from Luxembourg. Hydrobiologia..

[20] Gerecke R, Di Sabatino A, Cantonati M, Bertruzzi E, Spitale D (2007). Water mites (Hydrachnidia and Halacaridae) in spring habitats: a taxonomical and ecological perspective. The Spring habitat: biota and sampling methods.

[21] Rosati M, Cantonati M, Primicerio R, Rossetti G (2014). Biogeography and relevant ecological drivers in spring habitats: a review on ostracods of the Western Palearctic. Int Rev Hydrobiol..

[22] Cantonati M, Füreder L, Gerecke R, Jüttner I, Cox EJ (2012). Crenic habitats, hotspots for freshwater biodiversity conservation: toward an understanding of their ecology. Freshw Sci..

[23] Pešić V, Valdecasas AG, García-Jiménez R (2012). Simultaneous evidence for a new species of *Torrenticola* Piersing, 1896 (Acari: Hydrachnidia) from Montenegro. Zootaxa..

[24] Ding JH, Sun JL, Zhang X (2017). A new species of the water mite genus *Sperchon* Kramer, 1877 from China, with identifying *Sperchon rostratus* lundblad, 1969 through DNA barcoding (Acari, Hydrachnidia, Sperchontidae). ZooKeys..

[25] Pešić V, Smit H (2018). A checklist of water mites of Central Asia with description of six new species (Acari, Hydrachnidia) from Kyrgyzstan. Acarologia..

[26] Smit H (2018). A second species of the water mite genus Sinhaladwipabates Gledhill & Wiles, 1997 from Thailand (Acari: Hydrachnidia: Hygrobatidae). Ecol Montenegrina..

[27] Asadi M, Hinomoto N, Saboori A, Javan-Nikkhah M (2012). Genetic diversity in mitochondrial cytochrome *c* oxidase subunit I sequences of the water mite *Hygrobates fluviatilis* (Acari: Hydrachnidia: Hygrobatidae). Int J Acarol..

[28] Staalstedt J, Bergsten J, Ronquist F (2013). “Forms” of water mites (Acari: Hydrachnidia): intraspecific variation or valid species?. Ecol Evol..

[29] Pešić V, Asadi M, Cimpean M, Dabert M, Esen Y, Gerecke R (2017). Six species in one: evidence of cryptic speciation in the *Hygrobates fluviatilis* complex (Acariformes, Hydrachnidia, Hygrobatidae). Syst Appl Acarol..

[30] Pešić V, Broda Ł, Dabert M, Gerecke R, Martin P, Smit H (2019). Re-established after hundred years: definition of Hygrobates prosiliens Koenike, 1915, based on molecular and morphological evidence, and redescription of H. longipalpis (Hermann, 1804) (Acariformes, Hydrachnidia, Hygrobatidae). Syst Appl Acarology..

[31] Hebert PDN, Cywinska A, Ball SL, DeWaard JR (2003). Biological identifications through DNA barcodes. Proc Biol Sci..

[32] Copilaș-Ciocianu D, Zimța A-A, Petrusek A (2018). Integrative taxonomy reveals a new *Gammarus* species (Crustacea, Amphipoda) surviving in a previously unknown southeast European glacial refugium. J Zool Syst Evol Res..

[33] Lin X-L, Stur E, Ekrem T (2017). DNA barcodes and morphology reveal unrecognized species in Chironomidae (Diptera). Insect Syst Evol..

[34] Weiss M, Macher JN, Seefeldt MA, Leese F (2014). Molecular evidence for further overlooked species within the *Gammarus fossarum* complex (Crustacea: Amphipoda). Hydrobiologia..

[35] Montagna M, Mereghetti V, Lencioni V, Rossaro B (2016). Integrated taxonomy and DNA barcoding of alpine midges (Diptera: Chironomidae). PLoS ONE..

[36] Elbrecht V, Vamos EE, Meissner K, Aroviita J, Leese F (2017). Assessing strengths and weaknesses of DNA metabarcoding-based macroinvertebrate identification for routine stream monitoring. Methods Ecol Evol..

[37] Mächler E, Deiner K, Steinmann P, Altermatt F (2014). Utility of environmental DNA for monitoring rare and indicator macroinvertebrate species. Freshw Sci..

[38] Meyer CP, Paulay G (2005). DNA barcoding: error rates based on comprehensive sampling. PLoS Biol..

[39] Klopfstein S, Kropf C, Baur H (2016). *Wolbachia* endosymbionts distort DNA barcoding in the parasitoid wasp genus *Diplazon* (Hymenoptera: Ichneumonidae). Zool J Linn Soc..

[40] Smith MA, Bertrand C, Crosby K, Eveleigh ES, Fernandez-Triana J, Fisher BL (2012). *Wolbachia* and DNA barcoding insects: patterns, potential, and problems. PLoS ONE..

[41] Song H, Buhay JE, Whiting MF, Crandall KA (2008). Many species in one: DNA barcoding overestimates the number of species when nuclear mitochondrial pseudogenes are coamplified. Proc Natl Acad Sci USA.

[42] Haran J, Koutroumpa F, Magnoux E, Roques A, Roux G (2015). Ghost mtDNA haplotypes generated by fortuitous NUMTs can deeply disturb infra-specific genetic diversity and phylogeographic pattern. J Zool Syst Evol Res..

[43] Hazkani-Covo E, Zeller RM, Martin W (2010). Molecular poltergeists: mitochondrial DNA copies (numts) in sequenced nuclear genomes. PLoS Genet..

[44] Kimura M (1980). A simple method for estimating evolutionary rates of base substitutions through comparative studies of nucleotide sequences. J Mol Evol..

[45] Jinbo U, Kato T, Ito M (2011). Current progress in DNA barcoding and future implications for entomology. Entomol Sci..

[46] Collins RA, Boykin LM, Cruickshank RH, Armstrong KF (2012). Barcoding’s next top model: an evaluation of nucleotide substitution models for specimen identification. Methods Ecol Evol..

[47] Waugh J (2007). DNA barcoding in animal species: progress, potential and pitfalls. BioEssays..

[48] Hebert PD, Ratnasingham S, DeWaard JR (2003). Barcoding animal life: cytochrome *c* oxidase subunit 1 divergences among closely related species. Proc R Soc London Ser B Biol Sci..

[49] Sonnenberg R, Nolte A, Tautz D (2007). An evaluation of *LSU* rDNA D1-D2 sequences for their use in species identification. Front Zool..

[50] Lehmitz R, Decker P (2017). The nuclear 28S gene fragment D3 as species marker in oribatid mites (Acari, Oribatida) from German peatlands. Exp Appl Acarol..

[51] Mironov SV, Dabert J, Dabert M (2012). A new feather mite species of the genus *Proctophyllodes* Robin, 1877 (Astigmata: Proctophyllodidae) from the long-tailed tit *Aegithalos caudatus* (Passeriformes: Aegithalidae)-morphological description with DNA barcode data. Zootaxa..

[52] Vasquez AA, Qazazi MS, Fisher JR, Failla AJ, Rama S, Ram JL (2017). New molecular barcodes of water mites (Trombidiformes: Hydrachnidiae) from the Toledo Harbor region of Western Lake Erie, USA, with first barcodes for *Krendowskia* (Krendowskiidae) and *Koenikea* (Unionicolidae). Int J Acarol..

[53] Schizas NV, Street GT, Coull BC, Chandler GT, Quattro JM (1997). An efficient DNA extraction method for small metazoans. Mol Mar Biol Biotechnol..

[54] Böttger-Schnack R, Machida RJ (2011). Comparison of morphological and molecular traits for species identification and taxonomic grouping of oncaeid copepods. Hydrobiologia..

[55] Weigand AM (2013). New *Zospeum* species (Gastropoda, Ellobioidea, Carychiidae) from 980 m depth in the Lukina Jama-Trojama cave system (Velebit Mts., Croatia). Subteranean Biol..

[56] Bartsch I, Davids C, Deichsel R, Di Sapatino A, Gabrys G, Gerecke R, Gerecke R (2007). Chelicerata: Araneae, Acari I. Süsswasserfauna von Mitteleuropa.

[57] Di Sabatino A, Gerecke R, Gledhill T, Smit H, Gerecke R (2010). Chelicerata: acari II. Süsswasserfauna von Mitteleuropa.

[58] Gerecke R, Gledhill T, Pešić V, Smit H (2016). Süßwasserfauna Von Mitteleuropa, Bd 7/2-3. Chelicerata: Acari III.

[59] Folmer O, Black M, Hoeh W, Lutz R, Vrijenhoek R (1994). DNA primers for amplification of mitochondrial cytochrome *c* oxidase subunit I from diverse metazoan invertebrates. Mol Mar Biol Biotechnol..

[60] Messing J (1983). New M13 vectors for cloning. Methods Enzymol..

[61] Ivanova NV, Zemlak TS, Hanner RH, Hebert PDN (2007). Universal primer cocktails for fish DNA barcoding. Mol Ecol Notes..

[62] Kress WJ, Erickson DL, Barcodes DNA (2012). DNA barcodes: methods and protocols. Humana Press.

[63] Geneious. Geneious Prime. 2019. https://www.geneious.com. Accessed 13 Aug 2019.

[64] Katoh K, Standley DM (2013). MAFFT multiple sequence alignment software version 7: improvements in performance and usability. Mol Biol Evol..

[65] Talavera G, Castresana J (2007). Improvement of phylogenies after removing divergent and ambiguously aligned blocks from protein sequence alignments. Syst Biol..

[66] Castresana J (2000). Selection of conserved blocks from multiple alignments for their use in phylogenetic analysis. Mol Biol Evol..

[67] NCBI. BLASTn. 2019. https://blast.ncbi.nlm.nih.gov/Blast.cgi. Accessed 22 Aug 2019.

[68] Vaidya G, Lohman DJ, Meier R (2011). SeqenceMatrix: cladistics multi-gene datasets with character set and codon information. Cladistics..

[69] Darriba D, Posada D. ModelTest-NG. 2015. https://github.com/ddarriba/modeltest. Accessed 11 Feb 2019.

[70] Darriba D, Taboada GL, Doallo R, Posada D (2012). JModelTest 2: more models, new heuristics and parallel computing. Nat Methods..

[71] Darriba D, Taboada GL, Doallo R, Posada D (2011). ProtTest 3: fast selection of best-fit models of protein evolution. Bioinformatics..

[72] Miller MA, Pfeiffer W, Schwartz T. Creating the CIPRES science gateway for inference of large phylogenetic trees. In: Proceedings of the gateway computing environments workshop (GCE). New Orleans; 2010. p. 1–8.

[73] Kumar S, Stecher G, Li M, Knyaz C, Tamura K (2018). MEGA X: molecular evolutionary genetics analysis across computing platforms. Mol Biol Evol..

[74] Boyer S, Brown SDJ, Malumbres-Olarte J, Vink CJ, Cruickshank RH, Collins RA (2012). Spider: an R package for the analysis of species identity and evolution, with particular reference to DNA barcoding. Mol Ecol Resour..

[75] R Core Team. R: A language and environment for statistical computing. Vienna: R Foundation for Statistical Computing; 2017.

[76] Puillandre N, Lambert A, Brouillet S, Achaz G (2012). ABGD, automatic barcode gap discovery for primary species delimitation. Mol Ecol..

[77] Achaz G. ABGD HomePage. 2019. http://wwwabi.snv.jussieu.fr/public/abgd/. Accessed 20 Aug 2019.

[78] Ratnasingham S, Hebert PDN. The Barcode of Life Data System (http://www.barcodinglife.org). Mol Ecol Notes. 2007;7:355–64. 10.1111/j.1471-8286.2007.01678.x PMC1890991 18784790

[79] Ratnasingham S, Hebert PDN (2013). A DNA-based registry for all animal species: the Barcode Index Number (BIN) system. PLoS ONE..

[80] Barcode of Life Data Systems. BOLDsystems v4. 2019. http://boldsystems.org. Accessed 12 Jul 2019.

[81] Kozlov AM, Darriba D, Flouri T, Morel B, Stamatakis A (2019). RAxML-NG: a fast, scalable and user-friendly tool for maximum likelihood phylogenetic inference. Bioinformatics..

[82] Stamatakis A (2014). RAxML version 8: a tool for phylogenetic analysis and post-analysis of large phylogenies. Bioinformatics..

[83] Felsenstein J (1985). Confidence limits on phylogenies: an approach using the bootstrap. Evolution..

[84] Darling A, Ronquist F, Ayres DL, Larget B, Liu L, Teslenko M (2012). MrBayes 3.2: efficient Bayesian phylogenetic inference and model choice across a large model space. Syst Biol..

[85] Altekar G, Dwarkadas S, Huelsenbeck JP, Ronquist F (2004). Parallel Metropolis coupled Markov chain Monte Carlo for Bayesian phylogenetic inference. Bioinformatics..

[86] Douady CJ, Delsuc F, Boucher Y, Doolittle WF, Douzery EJP (2003). Comparison of Bayesian and maximum likelihood bootstrap measures of phylogenetic reliability. Mol Biol Evol..

[87] Dabert M, Proctor H, Dabert J (2016). Higher-level molecular phylogeny of the water mites (Acariformes: Prostigmata: Parasitengonina: Hydrachnidiae). Mol Phylogenet Evol..

[88] Rambaut A. FigTree. 2019. http://tree.bio.ed.ac.uk/software/figtree. Accessed 12 Jul 2019.

[89] Huson DH, Scornavacca C (2012). Dendroscope 3: an interactive tool for rooted phylogenetic trees and networks. Syst Biol..

[90] Hebert PDN, Gregory TR (2005). The promise of DNA barcoding for taxonomy. Syst Biol..

[91] Baird DJ, Hajibabaei M (2012). Biomonitoring 2.0: a new paradigm in ecosystem assessment made possible by next-generation DNA sequencing. Mol Ecol..

[92] De Rojas M, Doña J, Jovani R, Dimov I, Zurita A, Callejón R (2018). Evidence of cryptic species in the genus *Tinaminyssus* (Acari: Rhinonyssidae) based on morphometrical and molecular data. Exp Appl Acarol..

[93] Stryjecki R, Bańkowska A, Gryzińska M, Sarnacka E, Rutkowska M, Zawal A (2016). The use of molecular techniques in the taxonomy of water mites (Hydrachnidia, Acari). Acta Biol..

[94] Martin P, Dabert M, Dabert J (2010). Molecular evidence for species separation in the water mite *Hygrobates nigromaculatus* Lebert, 1879 (Acari, Hydrachnidia): evolutionary consequences of the loss of larval parasitism. Aquat Sci..

[95] Collins RA, Cruickshank RH (2013). The seven deadly sins of DNA barcoding. Mol Ecol Resour..

[96] Zhang AB, Muster C, Liang HB, Zhu CD, Crozier R, Wan P (2012). A fuzzy-set-theory-based approach to analyse species membership in DNA barcoding. Mol Ecol..

[97] García-Jiménez R, Horreo JL, Valdecasas AG (2017). Minimal barcode distance between two water mite species from Madeira Island: a cautionary tale. Exp Appl Acarol..

[98] Yu G, Rao D, Matsui M, Yang J (2017). Coalescent-based delimitation outperforms distance-based methods for delineating less divergent species: the case of *Kurixalus odontotarsus* species group. Sci Rep..

[99] Liu XF, Yang CH, Han HL, Ward RD, Zhang AB (2014). Identifying species of moths (Lepidoptera) from Baihua Mountain, Beijing, China, using DNA barcodes. Ecol Evol..

[100] Klimov PB, Skoracki M, Bochkov AV (2019). *Cox*1 barcoding *versus* multilocus species delimitation: validation of two mite species with contrasting effective population sizes. Parasit Vectors..

[101] Weigand H, Weiss M, Cai H, Li Y, Yu L, Zhang C (2017). Deciphering the origin of mito-nuclear discordance in two sibling caddisfly species. Mol Ecol..

[102] Toews DPL, Brelsford A (2012). The biogeography of mitochondrial and nuclear discordance in animals. Mol Ecol..

[103] Edwards S, Bensch S (2009). Looking forwards or looking backwards in avian phylogeography? A comment on Zink and Barrowclough 2008. Mol Ecol..

[104] Zink RM, Barrowclough GF (2008). Mitochondrial DNA under siege in avian phylogeography. Mol Ecol..

[105] Arabi J, Judson MLI, Deharveng L, Lourenço WR, Cruaud C, Hassanin A (2012). Nucleotide composition of CO1 sequences in Chelicerata (Arthropoda): detecting new mitogenomic rearrangements. J Mol Evol..

[106] Elbrecht V, Leese F (2017). Validation and development of COI metabarcoding primers for freshwater macroinvertebrate bioassessment. Front Environ Sci..

[107] Leray M, Yang JY, Meyer CP, Mills SC, Agudelo N, Ranwez V (2013). A new versatile primer set targeting a short fragment of the mitochondrial COI region for metabarcoding metazoan diversity: application for characterizing coral reef fish gut contents. Front Zool..

[108] Deagle BE, Jarman SN, Coissac E, Pompanon F, Taberlet P (2014). DNA metabarcoding and the cytochrome c oxidase subunit I marker: not a perfect match. Biol Lett..

[109] Vamos E, Elbrecht V, Leese F (2017). Short COI markers for freshwater macroinvertebrate metabarcoding. Metabarcod Metagenom.

[110] Brandon-Mong G-J, Gan H-M, Sing K-W, Lee P-S, Lim P-E, Wilson J-J (2015). DNA metabarcoding of insects and allies: an evaluation of primers and pipelines. Bull Entomol Res..

[111] Zhang GK, Chain FJJ, Abbott CL, Cristescu ME (2018). Metabarcoding using multiplexed markers increases species detection in complex zooplankton communities. Evol Appl..

[112] Elbrecht V, Taberlet P, Dejean T, Valentini A, Usseglio-Polatera P, Beisel J-N (2016). Testing the potential of a ribosomal 16S marker for DNA metabarcoding of insects. PeerJ..

[113] Wiȩcek M, Martin P, Lipinski A (2013). Water mites as potential long-term bioindicators in formerly drained and rewetted raised bogs. Ecol Indic..

[114] Goldschmidt T (2016). Water mites (Acari, Hydrachnidia): powerful but widely neglected bioindicators—a review. Neotrop Biodivers..

[115] Miccoli FP, Lombardo P, Cicolani B (2013). Indicator value of lotic water mites (Acari: Hydrachnidia) and their use in macroinvertebrate-based indices for water quality assessment purposes. Knowl Manag Aquat Ecosyst..

[116] Pešić V, Smit H (2016). Evidence of cryptic and pseudocryptic speciation in *Brachypodopsis baumi* species complex (Acari, Hydrachnidia, Aturidae) from Borneo, with description of three new species. Syst Appl Acarol..

[117] Pešić V, Smit H (2017). *Neumania kyrgyzica* sp. nov. a new water mite from Kyrgyzstan based on morphological and molecular data (Acari, Hydrachnidia: Unionicolidae). Syst Appl Acarol..

[118] Wiecek M, Martin P, Gabka M (2013). Distribution patterns and environmental correlates of water mites (Hydrachnidia, Acari) in peatland microhabitats. Exp Appl Acarol..

