# Supplementary figures and images for: Hidden biodiversity revealed by integrated morphology and genetic species delimitation of spring dwelling water mite species (Acari, Parasitengona: Hydrachnidia)
Source: Parasit Vectors. 2019 Oct 21;12:492. doi: 10.1186/s13071-019-3750-y (PMC6805402; doi:10.1186/s13071-019-3750-y)

**A**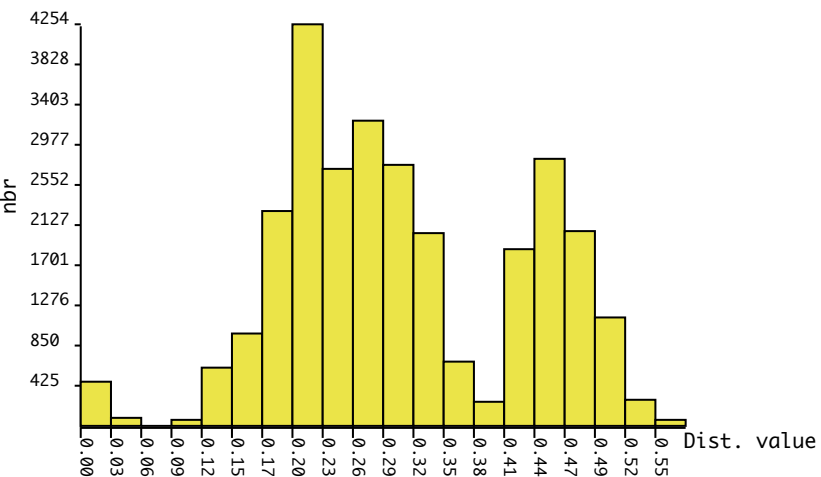**B**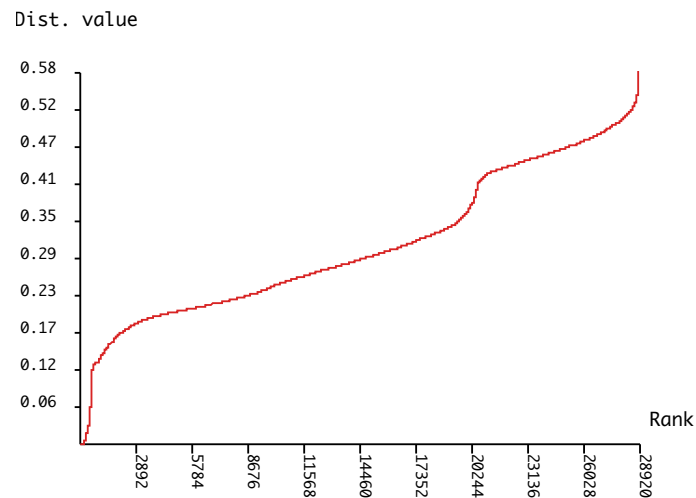**C**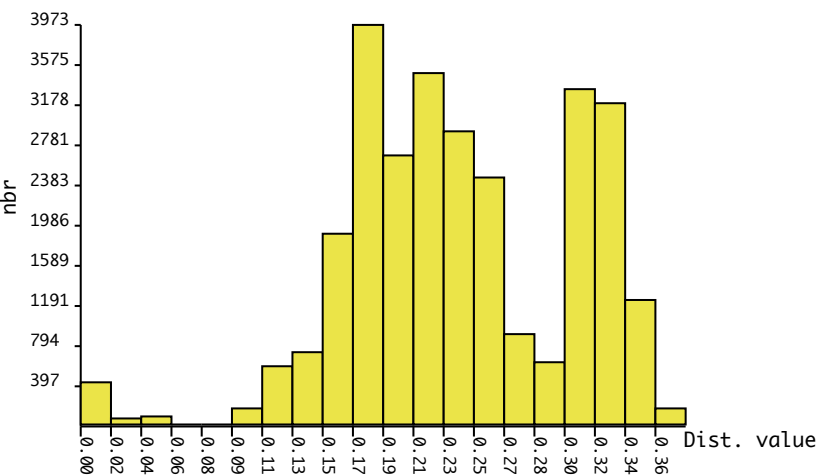**D**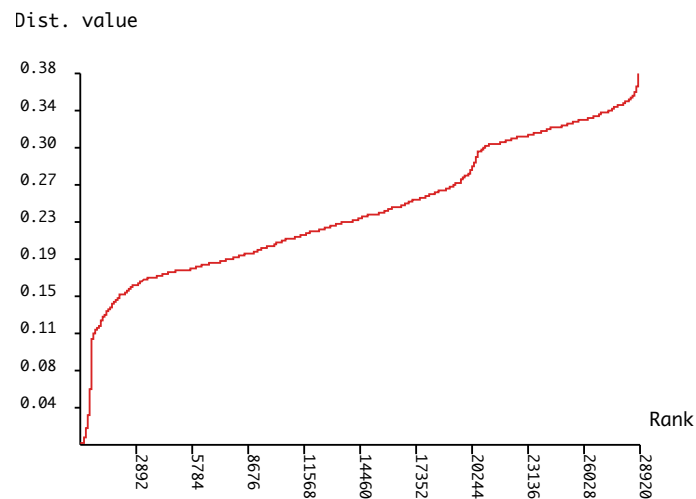

Supplement: Supplementary file 2 — Additional file 2: Figure S1. ABGD output plots of the cox1 K2P (A&B) and p-distances (C&D). [file 13071_2019_3750_MOESM2_ESM.pdf]

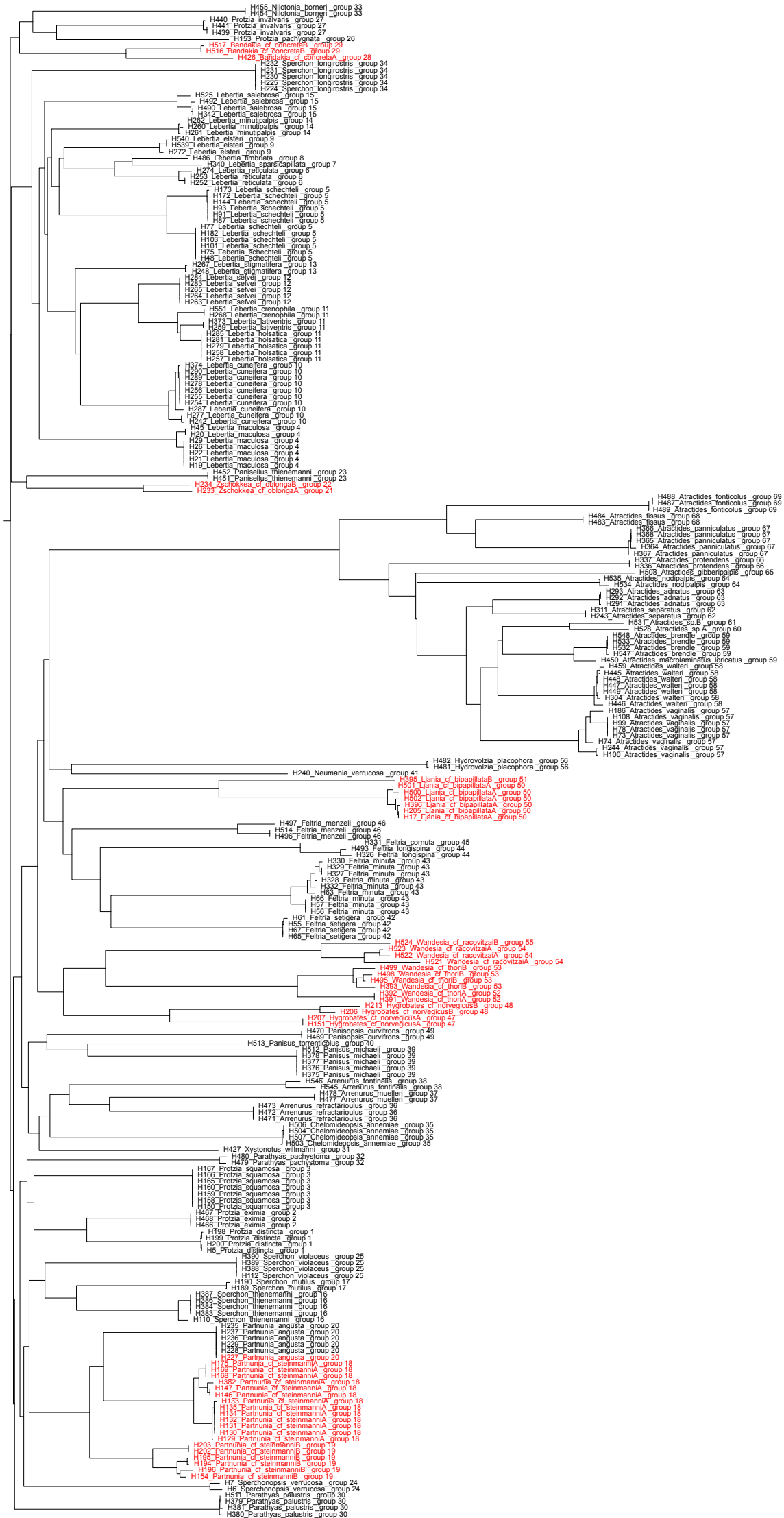

Supplement: Supplementary file 3 — Additional file 3: Figure S2. Initial partition ABGD cox1 output tree. Shows individuals grouped as putative species delimited by the ABGD method. Clades indicating more species than the a-priori morphospecies are marked in red. [file 13071_2019_3750_MOESM3_ESM.pdf]

**A**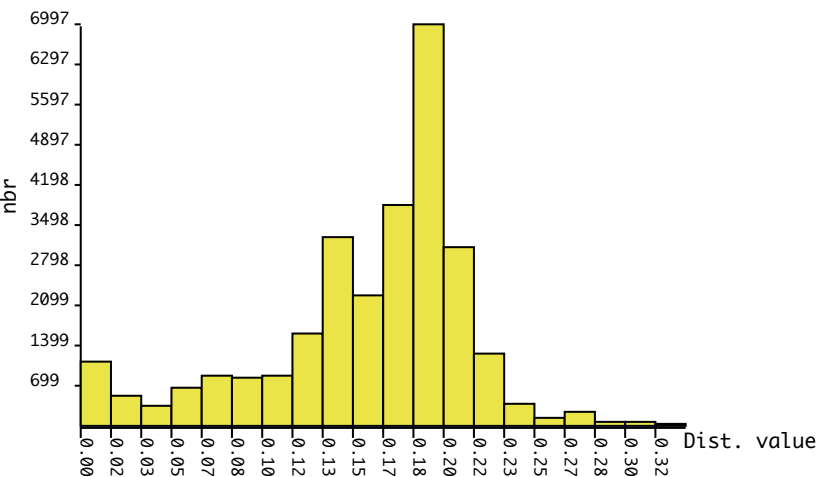**B**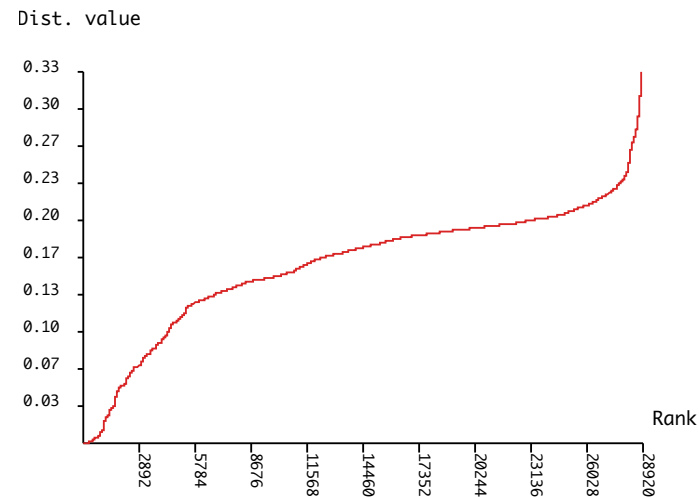**C**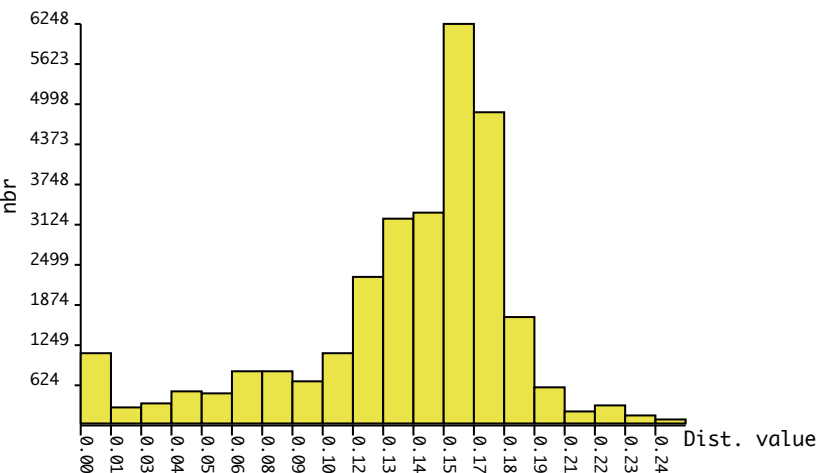**D**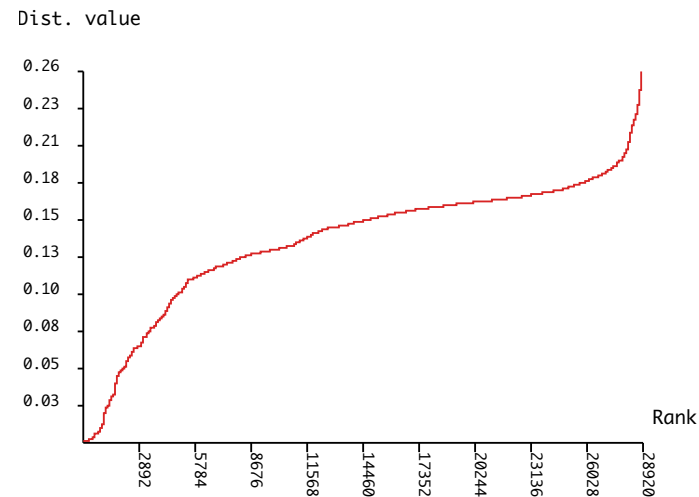

Supplement: Supplementary file 5 — Additional file 5: Figure S4. ABGD output plots of the 28S K2P (A&B) and p-distances (C&D). [file 13071_2019_3750_MOESM5_ESM.pdf]

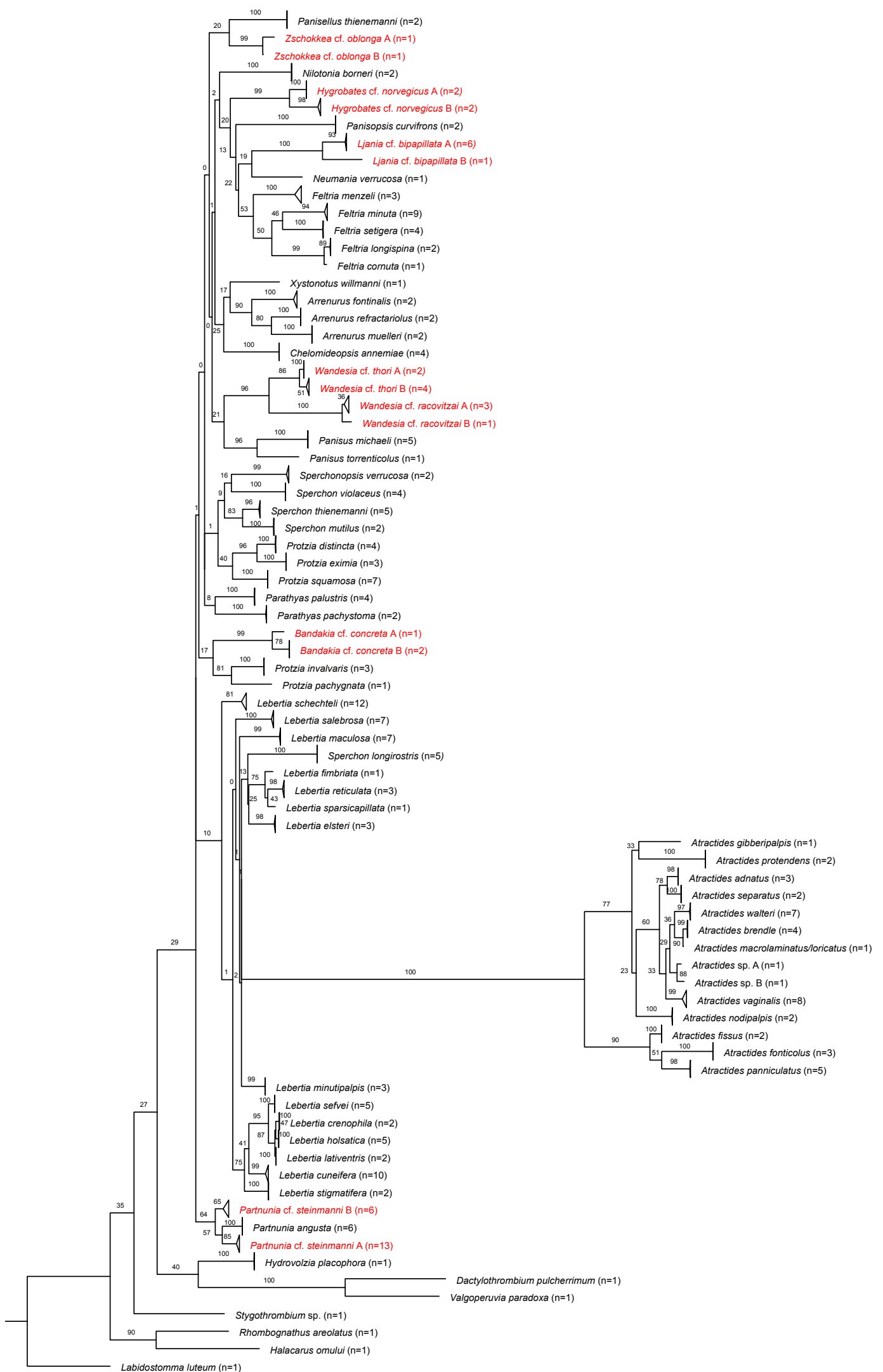

Supplement: Supplementary file 8 — Additional file 8: Figure S5. cox1 RAxML-NG Maximum Likelihood tree with support values. Clades indicating more species than the a-priori morphospecies are marked in red. [file 13071_2019_3750_MOESM8_ESM.pdf]

Hygrobatoidea

Arrenuroidea

Lebertioidea

Hydryphantoidea

Hydrovolzioidea

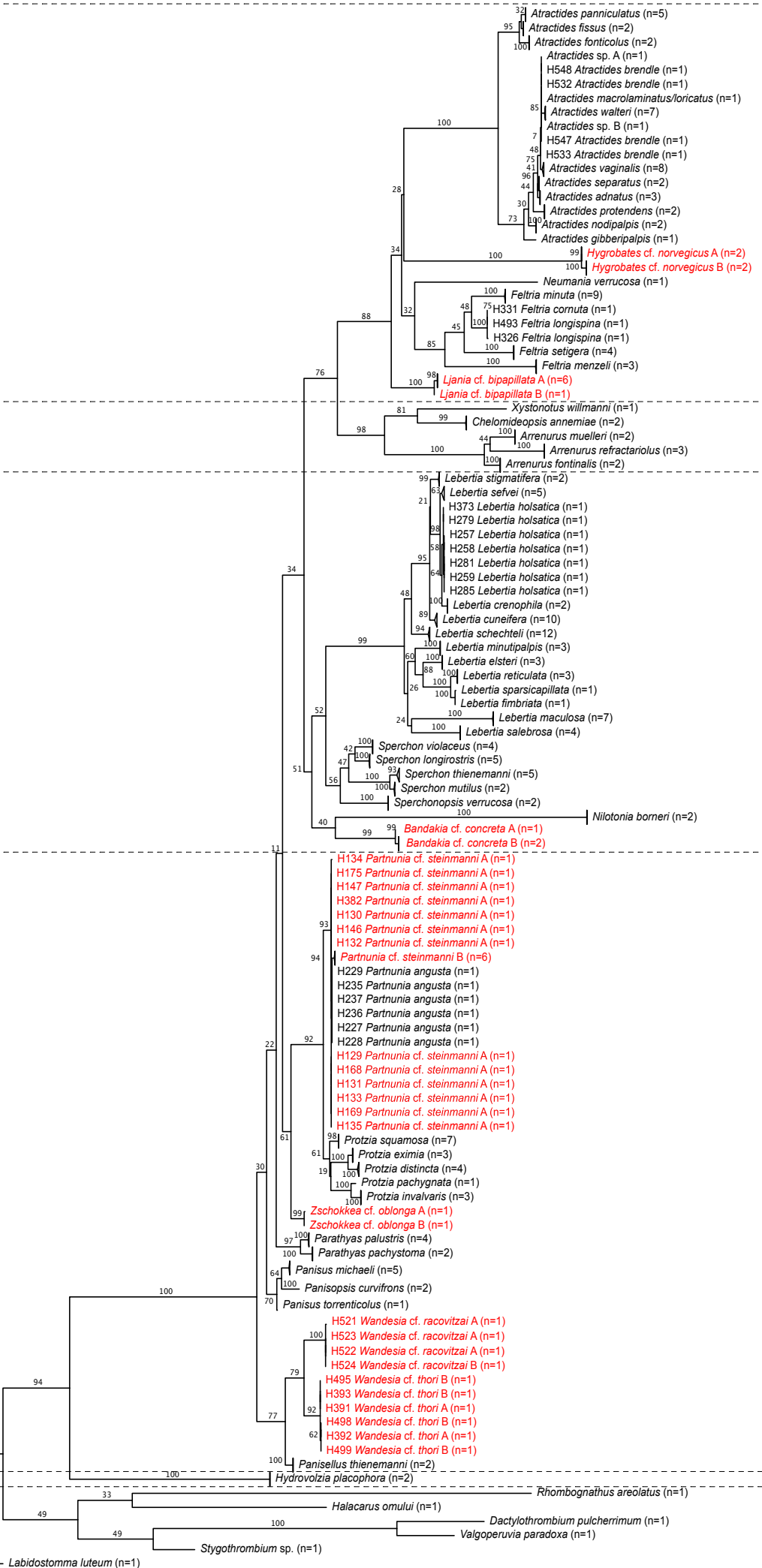

0.2

Supplement: Supplementary file 9 — Additional file 9: Figure S6. 28S RAxML-NG Maximum Likelihood tree with support values. Clades indicating more species than the a-priori morphospecies are marked in red. [file 13071_2019_3750_MOESM9_ESM.pdf]
