# Supplementary material for: Hidden biodiversity revealed by integrated morphology and genetic species delimitation of spring dwelling water mite species (Acari, Parasitengona: Hydrachnidia)
Source: Parasit Vectors. 2019 Oct 21;12:492. doi: 10.1186/s13071-019-3750-y (PMC6805402; doi:10.1186/s13071-019-3750-y)
Supplement: Supplementary file 4 — Additional file 4: Figure S3. Recursive partition ABGD cox1 output tree. Shows individuals grouped as putative species delimited by the ABGD method. Clades indicating more species than the a-priori morphospecies are marked in red and differences to the initial partition in blue. [file 13071_2019_3750_MOESM4_ESM.pdf]

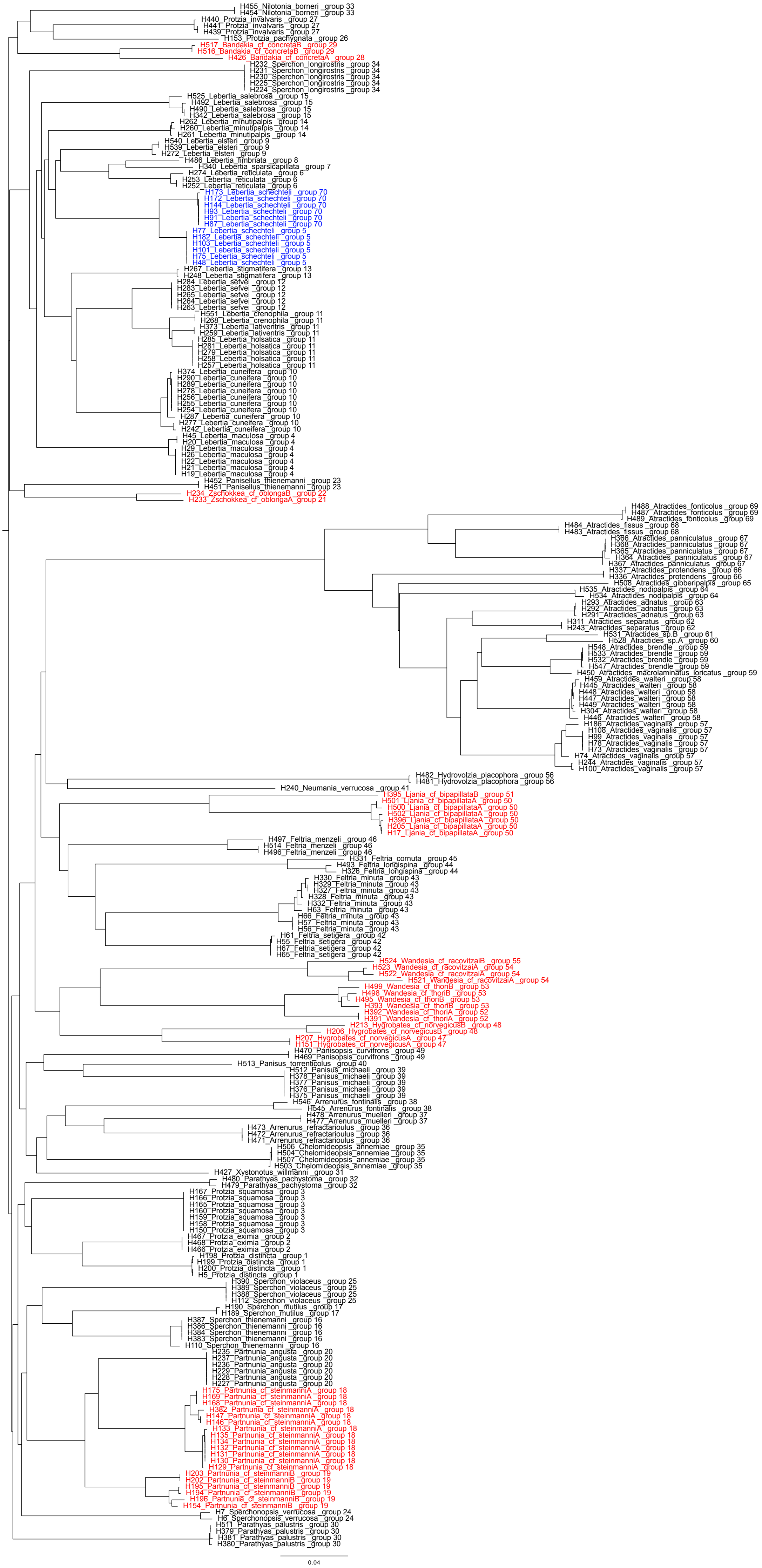
H455_Nilotonia_borneri _group 33
H454_Nilotonia_borneri _group 33
H440_Protzia_invalvaris _group 27
H441_Protzia_invalvaris _group 27
H439_Protzia_invalvaris _group 27
H153_Protzia_pachygnata _group 26
H517_Bandakia_cf_concretaB _group 29
H516_Bandakia_cf_concretaB _group 29
H426_Bandakia_cf_concretaA _group 28
H232_Sperchon_longirostris _group 34
H231_Sperchon_longirostris _group 34
H230_Sperchon_longirostris _group 34
H225_Sperchon_longirostris _group 34
H224_Sperchon_longirostris _group 34
H525_Lebertia_salebrosa _group 15
H492_Lebertia_salebrosa _group 15
H490_Lebertia_salebrosa _group 15
H342_Lebertia_salebrosa _group 15
H262_Lebertia_minutipalpis _group 14
H260_Lebertia_minutipalpis _group 14
H261_Lebertia_minutipalpis _group 14
H540_Lebertia_elsteri _group 9
H539_Lebertia_elsteri _group 9
H272_Lebertia_elsteri _group 9
H486_Lebertia_fimbriata _group 8
H340_Lebertia_sparsicapillata _group 7
H274_Lebertia_reticulata _group 6
H253_Lebertia_reticulata _group 6
H252_Lebertia_reticulata _group 6
H173_Lebertia_schechteli _group 70
H172_Lebertia_schechteli _group 70
H144_Lebertia_schechteli _group 70
H93_Lebertia_schechteli _group 70
H91_Lebertia_schechteli _group 70
H87_Lebertia_schechteli _group 70
H77_Lebertia_schechteli _group 5
H182_Lebertia_schechteli _group 5
H103_Lebertia_schechteli _group 5
H101_Lebertia_schechteli _group 5
H75_Lebertia_schechteli _group 5
H48_Lebertia_schechteli _group 5
H267_Lebertia_stigmatifera _group 13
H248_Lebertia_stigmatifera _group 13
H284_Lebertia_sefvei _group 12
H283_Lebertia_sefvei _group 12
H265_Lebertia_sefvei _group 12
H264_Lebertia_sefvei _group 12
H263_Lebertia_sefvei _group 12
H551_Lebertia_crenophila _group 11
H268_Lebertia_crenophila _group 11
H373_Lebertia_lativentris _group 11
H259_Lebertia_lativentris _group 11
H285_Lebertia_holsatica _group 11
H281_Lebertia_holsatica _group 11
H279_Lebertia_holsatica _group 11
H258_Lebertia_holsatica _group 11
H257_Lebertia_holsatica _group 11
H374_Lebertia_cuneifera _group 10
H290_Lebertia_cuneifera _group 10
H289_Lebertia_cuneifera _group 10
H278_Lebertia_cuneifera _group 10
H256_Lebertia_cuneifera _group 10
H255_Lebertia_cuneifera _group 10
H254_Lebertia_cuneifera _group 10
H287_Lebertia_cuneifera _group 10
H277_Lebertia_cuneifera _group 10
H242_Lebertia_cuneifera _group 10
H45_Lebertia_maculosa _group 4
H20_Lebertia_maculosa _group 4
H29_Lebertia_maculosa _group 4
H26_Lebertia_maculosa _group 4
H22_Lebertia_maculosa _group 4
H21_Lebertia_maculosa _group 4
H19_Lebertia_maculosa _group 4
H452_Panisellus_thienemanni _group 23
H451_Panisellus_thienemanni _group 23
H234_Zschokkea_cf_oblongaB _group 22
H233_Zschokkea_cf_oblongaA _group 21
H488_Atractides_fonticolus _group 69
H487_Atractides_fonticolus _group 69
H489_Atractides_fonticolus _group 69
H484_Atractides_fissus _group 68
H483_Atractides_fissus _group 68
H366_Atractides_panniculatus _group 67
H368_Atractides_panniculatus _group 67
H365_Atractides_panniculatus _group 67
H364_Atractides_panniculatus _group 67
H367_Atractides_panniculatus _group 67
H337_Atractides_protendens _group 66
H336_Atractides_protendens _group 66
H508_Atractides_gibberipalpis _group 65
H535_Atractides_nodipalpis _group 64
H534_Atractides_nodipalpis _group 64
H293_Atractides_adnatus _group 63
H292_Atractides_adnatus _group 63
H291_Atractides_adnatus _group 63
H311_Atractides_separatus _group 62
H243_Atractides_separatus _group 62
H531_Atractides_sp.B _group 61
H528_Atractides_sp.A _group 60
H548_Atractides_brendle _group 59
H533_Atractides_brendle _group 59
H532_Atractides_brendle _group 59
H547_Atractides_brendle _group 59
H450_Atractides_macrolaminatus_loricatus _group 59
H459_Atractides_walteri _group 58
H445_Atractides_walteri _group 58
H448_Atractides_walteri _group 58
H447_Atractides_walteri _group 58
H449_Atractides_walteri _group 58
H304_Atractides_walteri _group 58
H446_Atractides_walteri _group 58
H186_Atractides_vaginalis _group 57
H108_Atractides_vaginalis _group 57
H99_Atractides_vaginalis _group 57
H78_Atractides_vaginalis _group 57
H73_Atractides_vaginalis _group 57
H74_Atractides_vaginalis _group 57
H244_Atractides_vaginalis _group 57
H100_Atractides_vaginalis _group 57
H482_Hydrovolzia_placophora _group 56
H481_Hydrovolzia_placophora _group 56
H240_Neumania_verrucosa _group 41
H395_Ljania_cf_bipapillataB _group 51
H501_Ljania_cf_bipapillataA _group 50
H500_Ljania_cf_bipapillataA _group 50
H502_Ljania_cf_bipapillataA _group 50
H396_Ljania_cf_bipapillataA _group 50
H205_Ljania_cf_bipapillataA _group 50
H17_Ljania_cf_bipapillataA _group 50
H497_Feltria_menzeli _group 46
H514_Feltria_menzeli _group 46
H496_Feltria_menzeli _group 46
H331_Feltria_cornuta _group 45
H493_Feltria_longispina _group 44
H326_Feltria_longispina _group 44
H330_Feltria_minuta _group 43
H329_Feltria_minuta _group 43
H327_Feltria_minuta _group 43
H328_Feltria_minuta _group 43
H332_Feltria_minuta _group 43
H63_Feltria_minuta _group 43
H66_Feltria_minuta _group 43
H57_Feltria_minuta _group 43
H56_Feltria_minuta _group 43
H61_Feltria_setigera _group 42
H55_Feltria_setigera _group 42
H67_Feltria_setigera _group 42
H65_Feltria_setigera _group 42
H524_Wandesia_cf_racovitzaiB _group 55
H523_Wandesia_cf_racovitzaiA _group 54
H522_Wandesia_cf_racovitzaiA _group 54
H521_Wandesia_cf_racovitzaiA _group 54
H499_Wandesia_cf_thoriB _group 53
H498_Wandesia_cf_thoriB _group 53
H495_Wandesia_cf_thoriB _group 53
H393_Wandesia_cf_thoriB _group 53
H392_Wandesia_cf_thoriA _group 52
H391_Wandesia_cf_thoriA _group 52
H213_Hygrobates_cf_norvegicusB _group 48
H206_Hygrobates_cf_norvegicusB _group 48
H207_Hygrobates_cf_norvegicusA _group 47
H151_Hygrobates_cf_norvegicusA _group 47
H470_Panisopsis_curvifrons _group 49
H469_Panisopsis_curvifrons _group 49
H513_Panisus_torrenticolus _group 40
H512_Panisus_michaeli _group 39
H378_Panisus_michaeli _group 39
H377_Panisus_michaeli _group 39
H376_Panisus_michaeli _group 39
H375_Panisus_michaeli _group 39
H546_Arrenurus_fontinalis _group 38
H545_Arrenurus_fontinalis _group 38
H478_Arrenurus_muelleri _group 37
H477_Arrenurus_muelleri _group 37
H473_Arrenurus_refractarioulus _group 36
H472_Arrenurus_refractarioulus _group 36
H471_Arrenurus_refractarioulus _group 36
H506_Chelomideopsis_annemiae _group 35
H504_Chelomideopsis_annemiae _group 35
H507_Chelomideopsis_annemiae _group 35
H503_Chelomideopsis_annemiae _group 35
H427_Xystonotus_willmanni _group 31
H480_Parathyas_pachystoma _group 32
H479_Parathyas_pachystoma _group 32
H167_Protzia_squamosa _group 3
H166_Protzia_squamosa _group 3
H165_Protzia_squamosa _group 3
H160_Protzia_squamosa _group 3
H159_Protzia_squamosa _group 3
H158_Protzia_squamosa _group 3
H150_Protzia_squamosa _group 3
H467_Protzia_eximia _group 2
H468_Protzia_eximia _group 2
H466_Protzia_eximia _group 2
H198_Protzia_distincta _group 1
H199_Protzia_distincta _group 1
H200_Protzia_distincta _group 1
H5_Protzia_distincta _group 1
H390_Sperchon_violaceus _group 25
H389_Sperchon_violaceus _group 25
H388_Sperchon_violaceus _group 25
H112_Sperchon_violaceus _group 25
H190_Sperchon_mutilus _group 17
H189_Sperchon_mutilus _group 17
H387_Sperchon_thienemanni _group 16
H386_Sperchon_thienemanni _group 16
H384_Sperchon_thienemanni _group 16
H383_Sperchon_thienemanni _group 16
H110_Sperchon_thienemanni _group 16
H235_Partnunia_angusta _group 20
H237_Partnunia_angusta _group 20
H236_Partnunia_angusta _group 20
H229_Partnunia_angusta _group 20
H228_Partnunia_angusta _group 20
H227_Partnunia_angusta _group 20
H175_Partnunia_cf_steinmanniA _group 18
H169_Partnunia_cf_steinmanniA _group 18
H168_Partnunia_cf_steinmanniA _group 18
H382_Partnunia_cf_steinmanniA _group 18
H147_Partnunia_cf_steinmanniA _group 18
H146_Partnunia_cf_steinmanniA _group 18
H133_Partnunia_cf_steinmanniA _group 18
H135_Partnunia_cf_steinmanniA _group 18
H134_Partnunia_cf_steinmanniA _group 18
H132_Partnunia_cf_steinmanniA _group 18
H131_Partnunia_cf_steinmanniA _group 18
H130_Partnunia_cf_steinmanniA _group 18
H129_Partnunia_cf_steinmanniA _group 18
H203_Partnunia_cf_steinmanniB _group 19
H202_Partnunia_cf_steinmanniB _group 19
H195_Partnunia_cf_steinmanniB _group 19
H194_Partnunia_cf_steinmanniB _group 19
H196_Partnunia_cf_steinmanniB _group 19
H154_Partnunia_cf_steinmanniB _group 19
H7_Sperchonopsis_verrucosa _group 24
H6_Sperchonopsis_verrucosa _group 24
H511_Parathyas_palustris _group 30
H379_Parathyas_palustris _group 30
H381_Parathyas_palustris _group 30
H380_Parathyas_palustris _group 30
0.04
